# Proteome Profiling of RNF213 Depleted Cells Reveals Nitric Oxide Regulator DDAH1 Antilisterial Activity

**DOI:** 10.3389/fcimb.2021.735416

**Published:** 2021-11-03

**Authors:** Lia Martina, Caroline Asselman, Fabien Thery, Katie Boucher, Louis Delhaye, Teresa M. Maia, Bart Dermaut, Sven Eyckerman, Francis Impens

**Affiliations:** ^1^ VIB-UGent Center for Medical Biotechnology, VIB, Ghent, Belgium; ^2^ Department of Biomolecular Medicine, Ghent University, Ghent, Belgium; ^3^ Center for Medical Genetics, Ghent University Hospital, Ghent, Belgium; ^4^ VIB Proteomics Core, VIB, Ghent, Belgium; ^5^ Cancer Research Institute Ghent (CRIG), Ghent University, Ghent, Belgium

**Keywords:** *Listeria*, bacterial infection, Moyamoya, nitric oxide, NO, mass spectrometry, proteomics, ISG15

## Abstract

RNF213 is a large, poorly characterized interferon-induced protein. Mutations in RNF213 are associated with predisposition for Moyamoya disease (MMD), a rare cerebrovascular disorder. Recently, RNF213 was found to have broad antimicrobial activity *in vitro* and *in vivo*, yet the molecular mechanisms behind this function remain unclear. Using mass spectrometry-based proteomics and validation by real-time PCR we report here that knockdown of RNF213 leads to transcriptional upregulation of MVP and downregulation of CYR61, in line with reported pro- and anti-bacterial activities of these proteins. Knockdown of RNF213 also results in downregulation of DDAH1, which we discover to exert antimicrobial activity against *Listeria monocytogenes* infection. DDAH1 regulates production of nitric oxide (NO), a molecule with both vascular and antimicrobial effects. We show that NO production is reduced in macrophages from RNF213 KO mice, suggesting that RNF213 controls *Listeria* infection through regulation of DDAH1 transcription and production of NO. Our findings propose a potential mechanism for the antilisterial activity of RNF213 and highlight NO as a potential link between RNF213-mediated immune responses and the development of MMD.

## Introduction

RNF213 is a giant 591 kDa human protein conserved in vertebrates. RNF213 is unique in combining a dynein-like ATPase core with a ubiquitin E3 ligase module (Kamada et al., 2011; Ahel et al., 2020) The ATPase core contains six AAA (ATPase associated with a variety of cellular activities) units, but different from other AAA ATPases the RNF213 ATPase core does not generate movement. Instead, it seems to behave as an elaborated molecular switch that is involved in the oligomerization of RNF213 on the surface of lipid droplets and likely also bacteria (Sugihara et al., 2019; Ahel et al., 2020; Otten et al., 2021; Thery et al., 2021). Additionally, the recent cryo-EM structure revealed that RNF213 does not depend on its naming RING domain to function as an E3 ubiquitin ligase (Ahel et al., 2020). RNF213 classifies as a new type of E3 ligase, employing an RZ finger presented by the C-terminal lobe of the E3 shell, promoting autoubiquitilation (Otten et al., 2021).

RNF213 has mainly been studied in the context of Moyamoya disease (MMD) (Scott and Smith, 2009). MMD is a rare cerebrovascular disorder that is characterized by progressive occlusion of the internal carotid artery and the outgrowth of a characteristic network of tiny compensatory vessels (Suzuki and Takaku, 1969; Weinberg et al., 2011; Fujimura et al., 2016). The etiology of the disease is unknown, but genome-wide association studies identified RNF213 as a major susceptibility gene for MMD. Polymorphisms in RNF213 are associated with MMD in East Asian and Caucasian populations (Kamada et al., 2011; Guey et al., 2017), with the R4810K variant present as a founder mutation in many East Asian patients (Liu et al., 2011; Koizumi et al., 2016). Interestingly, the presence of RNF213 polymorphisms is not enough to develop MMD, suggesting that additional environmental factors trigger disease onset (Yamada et al., 1997; Ueno et al., 2002). In line with this, RNF213 knockout mice do not spontaneously develop any vascular phenotype that resembles MMD (Sonobe et al., 2014; Kanoke et al., 2016). In contrast, knockdown or knockout of RNF213 in zebrafish results in abnormal vascular morphology, confirming a role for RNF213 in blood vessel formation (Liu et al., 2011). Interestingly, MMD polymorphisms cluster near the E3 module of RNF213, suggesting that MMD might result from a deregulated ubiquitination activity.

RNF213 is expressed at baseline levels in many tissues (Liu et al., 2011), but is upregulated by LPS, TNFα and interferon (IFN), supporting the idea that inflammatory and innate immune signals might act as potential environmental triggers for MMD (Kobayashi et al., 2015; Ohkubo et al., 2015; Bosch et al., 2020; Sarkar and Thirumurugan, 2020). RNF213 is present in the cytosol (Liu et al., 2011), but overexpression, oleic acid treatment (Sugihara et al., 2019), interferon or LPS stimulation (Bosch et al., 2020; Thery et al., 2021) target the protein to the surface of lipid droplets (LDs), organelles that regulate lipid storage but also act as intracellular innate immune hubs (Bosch et al., 2020). Overexpression of RNF213 stabilizes LDs, removing adipose triglyceride lipase (ATGL) from their surface and functioning as a positive regulator of fat storage in the cells (Sugihara et al., 2019). Interestingly, LD targeting of RNF213 requires a functional ATPase core (Sugihara et al., 2019) since overexpression of RNF213 variants that are defective in ATP binding or hydrolysis were diffused in the cytosol. Interestingly, the same variants were previously shown to be required for oligomerization of RNF213 (Morito et al., 2014), suggesting that LD translocation of RNF213 is associated with its oligomerization. We recently found that this is indeed the case and showed that RNF213 oligomerizes on the surface of lipid droplets upon IFN induction (Thery et al., 2021).

In line with its localization to LDs (Sugihara et al., 2019), RNF213 was found to be an important modulator of lipotoxicity (Piccolis et al., 2019). Depletion of RNF213 protects cells from palmitate-induced lipotoxicity and this is associated with decreased ubiquitination of members of the nuclear factor κB (NF-κB) pathway. It was shown that RNF213 was required to activate the NF-κB pathway upon palmitate treatment, again linking the protein to inflammation (Piccolis et al., 2019). This NF-κB-inducing function of RNF213 would, however, be negatively regulated by its ubiquitin ligase activity (Takeda et al., 2020). Upstream, RNF213 is negatively regulated by Protein tyrosine phosphatase 1B (PTP1B or PTPN1) and this was found to be important for hypoxia sensing and control of non-mitochondrial oxygen consumption in tumours (Banh et al., 2016). Also in adipocytes, RNF213 is regulated by PTP1B following TNFα stimulation, potentially mediated by PPAR-γ (Sarkar and Thirumurugan, 2020).

Recently, we discovered that RNF213 acts as an intracellular sensor for proteins modified by ISG15, a ubiquitin-like modification that is induced by IFN-β and that has a broad antimicrobial activity. We found that type-I IFN signalling induces the oligomerization of RNF213 on lipid droplets (LDs) and showed that this process requires ISG15 modification of RNF213 itself. Most interestingly, we demonstrated that RNF213, through its binding to ISG15, has a broad antimicrobial activity *in vitro* and *in vivo*, counteracting infection against *Listeria monocytogenes*, human respiratory syncytial virus (RSV), coxsackievirus (CV) B3 and herpes simplex virus 1 (HSV-1). Along with a striking co-localization of RNF213 with intracellular bacteria, these findings uncovered a new role of RNF213 as a key antimicrobial effector (Thery et al., 2021). Similarly, it was recently shown that RNF213 counteracts *Salmonella* through the ubiquitylation of LPS, independent of its naming RING domain, but instead relying on an RZ finger in the E3 shell (Otten et al., 2021). Although this study broadens the antimicrobial activity of RNF213 even further, it remains to be determined whether RNF213 also directly ubiquitylates cell wall components of Gram+ bacteria, like *Listeria*, for which the underlying mechanisms remain currently elusive.

To identify potential antimicrobial pathways regulated by RNF213, we here used mass spectrometry-based proteomics as an untargeted approach to monitor protein-level changes upon depletion of RNF213 by siRNA. We found that the absence of RNF213 leads to the upregulation of major vault protein (MVP) and the downregulation of cysteine-rich protein 61 (CYR61) and dimethylarginine dimethylaminohydrolase 1 (DDAH1). We found that DDAH1 counteracts infection by *Listeria monocytogenes*, likely *via* effects on NO production as a potential mechanism underlying the antilisterial activity of RNF213.

## Materials and Methods

### Antibodies

The following primary antibodies were used for immunoblotting: mouse polyclonal anti-DDAH1 (Thermo Fisher Scientific, H00023576-BO1P), rabbit polyclonal Anti-CYR61/CCN1 (Abcam, ab10760), mouse monoclonal anti-MVP (Thermo Fisher Scientific, MA5-13871), mouse monoclonal anti-ISG15 (F-9, sc-166755, Santa Cruz Biotechnology), rabbit polyclonal anti-RNF213 (HPA003347, Merck), rabbit polyclonal anti-tubulin-α antibody (#ab18251, Abcam), mouse monoclonal anti-FLAG-tag (M2, #F3165, Merck). Aforementioned primary antibodies were revealed using goat polyclonal anti-mouse IgG (IRDye^®^ 800CW, Li-COR), goat polyclonal anti-rabbit-IgG (IRDye^®^ 800CW, Li-COR), goat polyclonal anti-mouse-IgG (IRDye^®^ 680RD, Li-COR) or goat polyclonal anti-rabbit-IgG (IRDye^®^680RD, Li-COR), except for anti-RSV serum which was revealed with secondary anti-goat (#sc-2020, Santa Cruz biotechnology).

### Cell Culture

HeLa cells (ATCC^®^ CCL-2™) were purchased from ATCC and were maintained in MEM medium (#11095080, Thermo Fisher Scientific) supplemented with 10% FBS, 1% GlutaMAX (#35050038, Thermo Fisher Scientific), 1% non-essential amino acids (#11140035, Thermo Fisher Scientific), 1% sodium pyruvate (#11360039, Thermo Fisher Scientific) and 1% HEPES (#15630056, Thermo Fisher Scientific).

All cell lines used tested negative for presence of mycoplasma using a mycoplasma PCR detection kit (Minerva Biolabs, MIN-11-1100).

HCT116 cells (ATCC^®^ CCL-247™) were purchased from ATCC and maintained in McCoy’s 5a Medium (#16600082, Thermo Fisher Scientific) supplemented with 10% FBS.

Bone marrow-derived macrophages (BMDMs) were obtained from Female and Male C57BL/6 mice (RNF213+/+ or RNF213−/−) between 8 and 12 weeks of age. Bone marrow cells were isolated from femurs, and pooled per 3 mice. Isolated bone marrow cells were cultured for 7 days at a density of 10^5^ cells/mL in DMEM/F-12 medium, supplemented with 10% FBS, 10 units/ml penicillin, 10 μg/mL streptomycin, and 20 ng/mL murine M-CSF at 37°C in a humidified atmosphere with 5% CO_2_. On day 3, fresh medium containing 40 ng/ml M-CSF was added. Cells were further differentiated for 4 days in M-CSF-containing medium. BMDMs were isolated from animals housed in an animal facility that operates under the Flemish Government License Number LA1400536. All experiments were done under conditions specified by law and authorized by the UGent Institutional Ethical Committee on Experimental Animals.

### siRNA Transfection

A commercially available siRNA pool was used to knock down human DDAH1 (#M-008528-00-0005, GEHealthcare Dharmacon), human CYR61 (#L-0004263-00-0005, GE Healthcare Dharmacon), human MVP (#M-004984-00-0005,GE Healthcare Dharmacon) and a scramble siRNA was used as a control (#D-001210-01-05, GE Healthcare Dharmacon). To knock down RNF213 for proteomics analysis, siRNAs (#M-023324-02, GE Healthcare Dharmacon) were transfected with DharmaFECT transfection reagent (#T-2001-02, GE Healthcare Dharmacon) according to the instructions of the manufacturer. For the *Listeria* infection assays, siRNAs were transfected with DharmaFECT transfection reagent (#T-2001-02, GE Healthcare Dharmacon) according to the instructions of the manufacturer. In these experiments, a reverse siRNA transfection protocol was adopted to knockdown the expression of ISG15 and RNF213 genes in HeLa cells prior to *Listeria* infection. Immunoblotting assays were conducted to confirm reduction of protein expression levels.

### Plasmids Transfection

Plasmid transfection was performed with Polyethylenimine (PEI, #23966-1, Polysciences) as transfection reagent at a ratio PEI/cDNA of 5:1 (w/w). Plasmids were used at a final concentration of 1 μg DNA/500,000 HeLa cells. The following plasmids were used: pSVsport (Mock plasmid) (Bio connect life sciences), FLAG-DDAH1 (Bio connect life sciences), FLAG-CYR61/CCN1 (Bio connect life sciences), FLAG-MVP (Bio connect life sciences).

### SDS-PAGE and Immunoblotting

Cells were lysed in 2x Laëmmli buffer containing 125 mM Tris-HCl pH 6.8, 4% SDS, 20% glycerol, 0,004% Bromophenol blue supplemented with 20 mM DTT. Protein samples were boiled for 5 min at 95°C and sonicated prior to SDS-PAGE. Samples were loaded on 4-20% polyacrylamide gradient gels (#M42015, Genescript), 4–15% Mini-PROTEAN TGX Gels (#4561084, Biorad), 3-8% Criterion XT tris-acetate gel (#3450130, Biorad) or 4-15% Criterion TGX gel (#5671083, Biorad) according to the guidelines of the manufacturer. For detection of RNF213, proteins were separated on a 3-8% Criterion XT tris-acetate gel (#3450130, Biorad) or 4-15% Criterion TGX gel (#5671083, Biorad) according to the instructions of the manufacturer. Proteins were transferred to PVDF membrane (#IPFL00010, Merck) for 3 hours at 60 V with Tris/Boric acid buffer at 50 mM/50 mM. Membranes were blocked for 1 hour at room temperature (RT) with blocking buffer (#927-50000, LI-COR) and incubated with primary antibodies overnight at 4°C diluted to 1:1000 in TBS. The next day, membranes were washed three times for 15 min with TBS-Tween 0.1% (v/v) buffer and further incubated at RT for 1 h with the appropriate secondary antibody. Membranes were washed twice with TBS-tween 0.1% and once with TBS prior to detection. Immunoreactive bands were visualized on a LI-COR-Odyssey infrared scanner (Li-COR).

### *In Vitro* Infection With *Listeria monocytogenes*

*Listeria monocytogenes* EGD (BUG600 strain) was grown in brain heart infusion (BHI) medium at 37°C. *Listeria* were cultured overnight and then subcultured 1:10 in BHI medium for 2h at 37°C. Bacteria were washed three times in PBS and resuspended in medium without FBS prior to infection. HeLa cells were grown in 6-well plates at 37°C in 5% CO_2_ humidified atmosphere and infected with *Listeria* at a multiplicity of infection (MOI) of 25 or 10. Right after infection, plates were centrifuged at 1,000 g for 1 min followed by incubation for 1h at 37°C in 5% CO_2_ humidified atmosphere to allow entry of the bacteria. Afterwards, cells were washed two times with PBS and then grown in MEM medium with 10% FBS, 1% GlutaMAX, 1% non-essential amino acids, 1% sodium pyruvate, 1% hepes, supplemented with 40 μg/ml of gentamicin to kill extracellular bacteria, and incubated at 37°C in 5% CO_2_ humidified atmosphere for 24h or lysed immediately. For immunoblotting, infected cells were washed with ice-cold PBS, lysed in 2x Laëmmli buffer and further processed as described above. To count the number of bacteria inside cells, HeLa cells were washed with medium without gentamycin and lysed with milliQ water (200 microliter per well) to release intracellular bacteria. The water was pipetted up and down 5 times to completely disrupt the HeLa cells. Colony Forming Units (CFUs) were determined by serial dilution (1:10, 1:100, 1:1000, 1:10000) in milliQ water. 20 μl (out of 200 μl) of each dilution was plated in one quadrant of a 10 cm BHI agar plate. Each plate was divided into 4 quadrants, plating 4 dilutions per plate. The plates were transferred to an incubator at 37°C, to let the bacterial colonies grow. After 48h, colonies on the plates were counted (at least 100). Each experiment was performed in biological triplicate, each time with 3 technical replicates per condition. HCT116 cells were infected in 6-well plates analogue to HeLa cells, using McCoy’s 5A medium (Dulbecco) with 10% FBS (during cell culture), 2% FBS (during 1h bacterial entry), or 10% FBS and 40 μg/ml of gentamicin (during 24h infection). Experiments with HCT-116 cells were performed in biological duplicate, each time with 2 technical replicates per condition.

BMDMs were infected in 6-well plates analogue to HeLa cells (16h infection instead of 24h), using BMDM medium with 10% FBS (during cell culture), 2% FBS (during 1h bacterial entry) or 10% FBS and 40 μg/ml of gentamicin (during 16h infection). For BMDMs, experiments were performed in biological duplicate, each time with 4 technical replicates per condition.

### Proteomics Sample Preparation and LC-MS/MS Analysis

167,000 HeLa cells were seeded in 6-well plates and a commercially available siRNA pool was used to knockdown RNF213 (#M-023324-02, GE Healthcare Dharmacon). As control siRNA treatment, a pool of four scrambled siRNAs (#D-001206-13, GE Healthcare Dharmacon) was used. siRNAs were transfected with DharmaFECT transfection reagent (#T-2001-02, GE Healthcare Dharmacon) according to the instructions of the manufacturer. The next day, cells were either treated with 10 ng/mL of interferon-β (#11343524, Immunotools), or left untreated, for 24h. There were four biological conditions consisting of four replicates to generate 16 samples. Cells were homogenized in 100 µL 9 M urea, 20 mM HEPES pH 8.0. Samples were sonicated by three pulses of 5 s at an amplitude of 30% and centrifuged for 15 min at 16,000 × g at RT. Proteins were reduced with 5 mM DTT for 30 min at 55°C. Alkylation was done with 10 mM chloroacetamide for 15 min at RT in the dark after which 5 mM DTT was added again to stop the alkylation reaction. Samples were diluted to 4 M urea and 100 µg protein of each sample was digested with 1 µg endopeptidase LysC (Promega) (1/100, w/w) for 4h at 37°C. Samples were further diluted to 2 M urea and proteins were digested with 1 µg trypsin (Promega) (1/100, w/w) overnight at 37°C. Peptides were desalted on reversed phase C18 OMIX tips (Agilent). Purified peptides were dried under vacuum in HPLC inserts and stored at −20°C until LC-MS/MS analysis.

Peptides were re-dissolved in 30 µL loading solvent A (0.1% TFA in water/ACN (98:2, v/v)) and after UV peptide quantitation (Maia et al., 2020) 2 µg was injected for LC-MS/MS analysis on an Ultimate 3000 RSLCnano system in-line connected to a Q Exactive HF mass spectrometer (Thermo Fisher). Trapping was performed at 10 μL/min for 4 min in loading solvent A on a 20 mm trapping column (100 μm internal diameter (I.D.), 5 μm beads, C18 Reprosil-HD, Dr. Maisch, Germany) after which peptides were separated on a 200 cm long micro pillar array column (µPAC™, PharmaFluidics) with C18-endcapped functionality. Peptides were eluted from the analytical column by a non-linear gradient from 2 to 55% solvent B (0.1% FA in water/acetonitrile (2:8, v/v)) over 120 min at a constant flow rate of 300 nL/min, followed by a 5 min wash with 97% solvent B. The column was then re-equilibrated with 98% solvent A for 20 min. The column temperature was kept constant at 40°C in a column oven (CoControl 3.3.05, Sonation). The mass spectrometer was operated in positive and data-dependent mode, automatically switching between MS and MS/MS acquisition for the 16 most abundant ion peaks. Full scan MS spectra were acquired in the Orbitrap (375–1,500 m/z, AGC target 3E6, maximum injection time 60 ms) with a resolution of 60,000 (at 200 m/z). The most intense ions (threshold >1.3E4, precursor charge state 2 to 6, dynamic exclusion time 12 s) were isolated in the trap with an isolation window of 1.5 Da. MS/MS spectra (200-2,000 m/z, AGC target 1E5, maximum injection time 80 ms, centroid data type) were acquired in the orbitrap at fixed first mass 145 m/z and at a resolution of 15,000 (at 200 m/z). Peptide match was set on “preferred” and isotopes were excluded. Fragmentation was performed at a normalized collision energy of 28%. The polydimethylcyclosiloxane background ion at 445.12003 Da was used for internal calibration (lock mass).

### Proteomics Data Analysis

**Figure 1:**
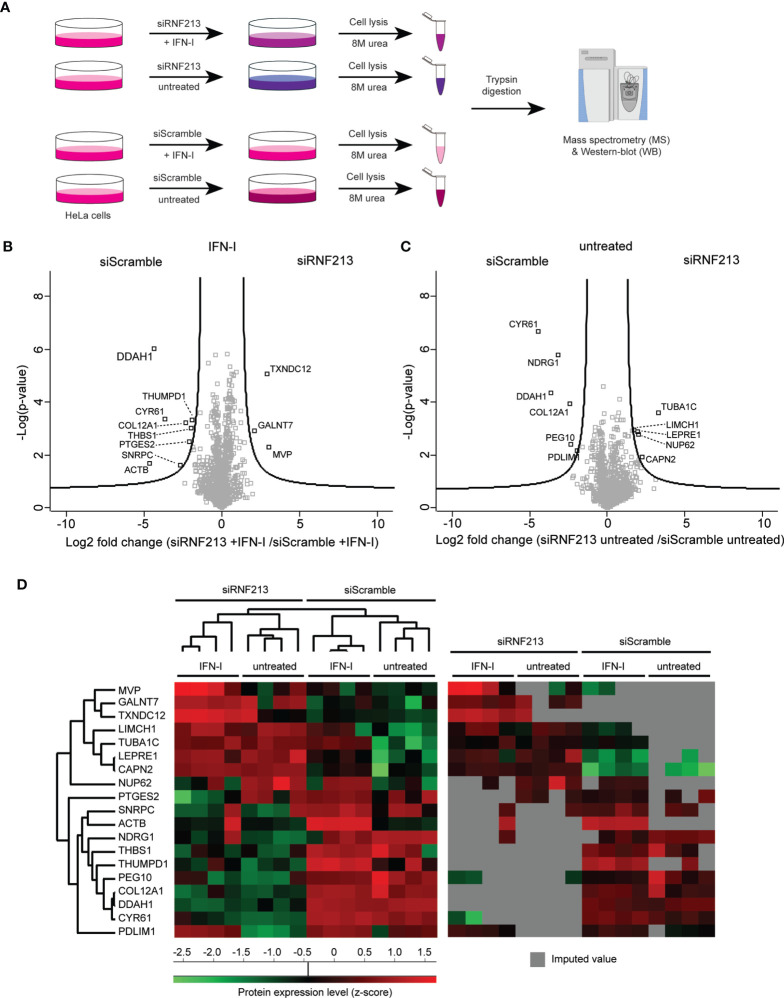
Proteome analysis of RNF213 depleted cells. **(A)** Experimental workflow describing the proteomics analysis of RNF213-depleted cells. Following 24h siRNA transfection, HeLa cells were treated with 10 ng/mL interferon-β (IFN-β) for 24h or left untreated prior to lysis in 8 M urea and trypsin digestion of the extracted proteins. Each condition was analysed in quadruplicate. Resulting peptides were analysed by LC-MS/MS analysis. Proteins were identified and quantified using the MaxQuant software followed by statistical analysis in Perseus. **(B, C)** Volcano plots showing the result of SAM t-tests available in Perseus software (FDR=0.05; S0=1) comparing the RNF213-depleted versus control cells that were either treated with IFN-β or left untreated. The fold change (in log_2_) of each protein is shown on the X-axis, while the statistical significance (−log P-value) is shown on the Y-axis. Proteins outside the curved lines represent differentially regulated proteins upon RNF213 depletion (**Supplementary Tables S3, S4**) **(D)** Proteins differentially regulated in the above volcano plots were displayed on a heat map after non-supervised hierarchical clustering. On the right side, the same heat map is shown with originally missing values coloured in gray.

Data analysis was performed with MaxQuant (version 1.6.11.0) using the Andromeda search engine with default search settings including a false discovery rate set at 1% on both peptide and protein level. All 16 raw spectral data files were searched together against the human reference proteome in Uniprot (downloaded from www.uniprot.org, database release from January 2020 containing 20,595 human protein sequences). Mass tolerance for precursor ions and fragment ions was set to 4.5 and 20 ppm, respectively, during the main search. Enzyme specificity was set as C-terminal to arginine and lysine (trypsin), also allowing cleavage at arginine/lysine–proline bonds with a maximum of two missed cleavages. Carbamidomethylation of cysteine residues was set as a fixed modification and oxidation of methionine residues and acetylation of protein N-termini were set as variable modifications. Matching between runs was disabled and only proteins with at least one unique peptide were retained to compile a list of 3,377 identified proteins. Proteins were quantified by the MaxLFQ algorithm integrated in the MaxQuant software with a minimum ratio count of one unique peptide. Further data analysis was performed with the Perseus software (version 1.6.2.3). The proteinGroups table from MaxQuant was loaded in Perseus and reverse database hits, potential contaminants and proteins only identified by site were removed. The MaxLFQ intensities were log_2_ transformed and replicate samples of each condition were grouped. Proteins with less than four valid values in at least one group were removed and missing values were imputed from a normal distribution around the detection limit to generate a list of 1,426 quantified proteins (**Supplementary Tables S1, S2**). To generate the volcano plots shown in **Figures 1B, C**, SAM t-tests (FDR = 0.05 and S0 = 1 cut-off values) (Tusher et al., 2001) were performed to compare the intensity of the proteins between the RNF213 knockdown and control conditions, either in the absence or presence of IFN type I. Significant hits are also shown in a heatmap in **Figure 1D** after non-supervised hierarchical clustering. The volcano plots and heatmap depicted in **Supplementary Figure 1** were generated similarly after comparing protein intensities between IFN-β treated and untreated samples, either with or without knockdown of RNF213 (**Supplementary Tables S1, S2**).

### Nitrite Concentration Measurements

BMDMs were seeded in clear low binding 96 well microtitrer plates (F-bottom, Greiner Cat nr M0812) at a density of 2x10^5^ cells per well in 200 µl BMDM medium containing 20 ng/ml M-CSF. LPS was added in the medium at final concentration of 100 ng/ml, as well as mouse IFN-γ (#12343534, immunotools) at final concentration of 10 ng/ml. Cells were cultured in these conditions for 24h. Nitrite concentration was measured using the Griess Reagent System (Promega, G2930) according to the protocol enclosed in the kit. In short: a Nitrite Standard Reference curve was prepared by serial dilution (100, 50, 25, 12.5, 6.25, 3.13 and 1.56 μM in triplicate) of the Nitrite standard enclosed in the kit. 50 μl of each experimental sample was added (8 technical replicates per sample) to wells. 50 μl of the sulfanilamide solution was dispensed to all wells, and incubated during 5 min, protected from light. 50 μL of NED solution was added to all wells and incubated during 5 min, protected from light. Absorbance was measured within 30 min at 537 nm on an Ensight instrument. GraphPad Prism was used for calibration curve fitting. The nitrite concentration in the experimental samples was determined in comparison to the Nitrite Standard reference curve. Two independent experiments were performed, each with 8 biological replicates per condition.

### Quantitative Reverse Transcription PCR Analysis

Total RNA was extracted from HeLa cells (5x10^6^ per biological sample) using NucleoSpin RNA, Mini kit for RNA purification (Machery-Nagel). Total RNA concentrations were measured by absorbance at 260 nm, and quality was assessed by A260/A280 ratios. cDNA synthesis was performed using the iScript cDNA synthesis kit (BioRad) using 500 ng RNA input. Target transcripts were amplified using primers listed in **Table 1**, using SensiFast SYBR No-ROX kit (BioLine) as described by the manufacturer’s instructions. Quantitative PCRs were assayed in duplo on a LightCycler480 (Roche) using the following cycling conditions: 1 cycle at 95°C for 5 min, 40 cycles at 95°C for 10 s, 60°C for 10 s and 72°C for 10 s, followed by a melting curve analysis to validate unique amplicons. Quantitation cycle (Cq) values for targets were analysed relative to Cq values for *SDHA*, *UBC*, *YWHAZ*, and *GAPDH* housekeeping genes. Housekeeping genes were selected using the geNorm algorithm as implemented in qbase+ (Hellemans et al., 2007) (Biogazelle). ΔCq of the target genes were further normalized relative to the scrambled siRNA control. All qPCR reactions were assayed in technical duplicate for each biological replicate sample. In total, 2 independent experiments were performed, each containing 4 biological replicates per condition.

**Table 1:** RT-qPCR primers used to amplify target transcripts.

Sequence Name	Sequence
DDAH1_Fwd	ACTCACTGTGCCTGACA
DDAH1_Rev	CAGTTCAGACATGCTCACGG
CCN1_Fwd	ATGGTCCCAGTGCTCAAAGA
CCN1_Rev	GGGCCGGTATTTCTTCACAC
MVP_Fwd	ATGAGTGGCTTTTCGAGGGA
MVP_Rev	CATTCTTCCCCTGTCACCCT
YWHAZ_Fwd	ACTTTTGGTACATTGTGGCTTCAA
YWHAZ_Rev	CCGCCAGGACAAACCAGTAT
SDHA_Fwd	TGGGAACAAGAGGGCATCTG
SDHA_ Rev	CCACCACTGCATCAAATTCATG
UBC_Fwd	ATTTGGGTCGCGGTTCTTG
UBC_Rev	TGCCTTGACATTCTCGATGGT
GAPDH_Fwd	TGCACCACCAACTGCTTAGC
GAPDH_ Rev	GGCATGCACTGTGGTCATGAG

### Statistics/Data Availability

Statistical analysis of the data was performed in GraphPad Prism v7.00 for Windows (GraphPad Software, La Jolla, USA). Data summaries are given as means ± SEM. Two-tailed unpaired t-tests were used for two-group comparisons. A p-value of 0.05 was considered as cut-off for statistical significance. The following notation for statistical significance was used: ns (p> 0.05), *(p ≤ 0.05), **(p ≤ 0.01), ***(p ≤ 0.001) **** (p ≤ 0.0001).

## Results

### Proteome Alterations Induced by RNF213 Knockdown

To elucidate the molecular mechanisms underlying the antimicrobial activity of RNF213 we set out to identify proteins regulated by RNF213 by untargeted mass spectrometry-based proteome analysis. To this end, we knocked down RNF213 by a pool of siRNAs (siRNF213) in HeLa cells, conditions that were previously shown to promote infection with *Listeria monocytogenes*, HSV-1 and CVB3 (Thery et al., 2021). Since RNF213 is upregulated by type-I interferon we also included interferon-β (IFN-β) treatment in our screen, leading to four experimental conditions each analysed in quadruplicate (**Figure 1A**). Upon siRNA and/or IFN-β treatment, cells were lysed and extracted proteins were digested with trypsin. The resulting peptide mixtures were analysed by liquid chromatography-tandem mass spectrometry (LC-MS/MS) in a labelfree proteomics workflow leading to the quantification of 1,426 proteins.

To check for proper interferon treatment, we first performed a pairwise comparison of protein intensities between untreated and interferon-β treated cells, either with or without knockdown of RNF213 (**Supplementary Figures 1A–C** and **Supplementary Tables S1, S2**). This analysis revealed the upregulation of 38 proteins by IFN-β, an induction that happened to a similar extent with and without knockdown of RNF213. As expected, the expression levels of well-known Interferon Stimulated Genes (ISGs) such as STAT1, MXA, ISG15 and IFIT1 (Schneider et al., 2014) were strongly increased upon interferon-β treatment (**Supplementary Figure 1D**). This similar response in RNF213 knockdown and control cells shows that depletion of RNF213 in HeLa cells does not lead to major disturbance of the type-I IFN signalling pathway.

To reveal potential downstream effectors of RNF213, we then performed a pairwise comparison of protein intensities in siRNF213-treated cells versus scramble siRNA-treated cells, either in the presence or absence of IFN-β. These comparisons led to 19 significantly regulated proteins (**Figures 1B, C** and **Supplementary Tables S3, S4**) that were displayed in a heatmap after non-supervised hierarchical clustering (**Figure 1D**). This heatmap revealed that most proteins were undetected in at least one experimental condition, suggesting strong up- and downregulation. Knockdown of RNF213 led to downregulation of eleven proteins, while eight proteins were upregulated. Interestingly, three proteins (MVP, GALNT7 and TXNDC12) were only upregulated in the presence of IFN-β.

### RNF213 Knockdown Downregulates DDAH1 and CYR61 at the Transcriptional Level

For further work we decided to focus on DDAH1 and CYR61 given their strong downregulation upon RNF213 knockdown and potential role in the host response to infection. Similar to RNF213, a previous genome-wide siRNA screen ranked DDAH1 and CYR61 as protective host factors during infection with *Listeria monocytogenes* (further referred to as *Listeria*), a facultative intracellular bacterial model pathogen (Kuhbacher et al., 2015) (**Supplementary Figure 2**). In line with this, CYR61 was recently identified as a pattern recognition receptor that opsonizes bacteria for clearance and activates inflammatory responses (Jun and Lau, 2020). DDAH1 has not directly been linked to infection, but as an important regulator of nitric oxide (NO) generation, the protein is an interesting target since NO plays an important role in immune defence as well as vascular regulation (Shiva, 2013; Hulin et al., 2020). Among the upregulated proteins, we further explored MVP since this protein is known to promote infection with *Listeria*. MVP is recruited to the surface of cytosolic *Listeria*, protecting the bacterium from clearance by autophagy (Dortet et al., 2012). We recently found that also RNF213 co-localizes with a subset of intracellular *Listeria*, however, in contrast to MVP, RNF213 strongly counteracts the bacterium.

**Figure 2:**
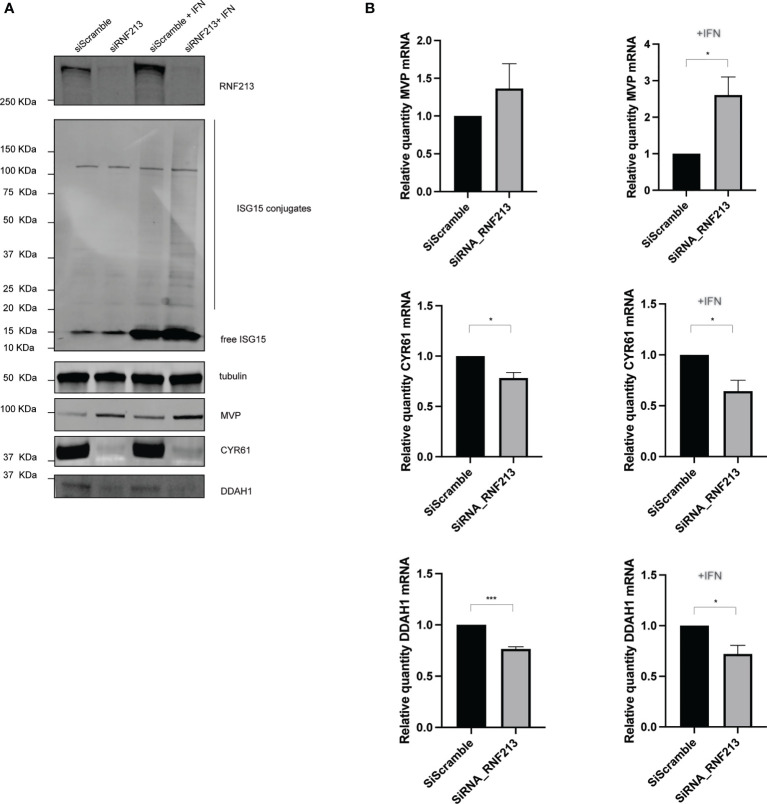
RNF213 knockdown downregulates DDAH1 and CYR61 at the transcript level. HeLa cells were treated with IFN-β in combination with knockdown of RNF213 as described in **Figure 1**. **(A)** Immunoblotting on the actual samples used for proteome analysis confirmed efficient knockdown of RNF213 and interferon-induced upregulation of free and conjugated ISG15. Immunoblots against DDAH1, CYR61 and MVP validated downregulation of DDAH1 and CYR61, and upregulation of MVP upon knockdown of RNF213. Tubulin was used as loading control. Densitometry and statistical analysis on two more biological repeats confirmed significant up- or downregulation of the immunoblotting bands (**Supplementary Figure 3**). **(B)** Real time qPCR upon knockdown of RNF213 showed significant upregulation of MVP in the presence of IFN-β and significant downregulation of CYR61 and DDAH1 transcripts in the presence and absence of IFN-β. Expression was normalized to housekeeping genes and shown relative to control (siScramble) populations set to 1. Data represents 3 biological replicates with 4 technical replicates per condition. (AVG ± SEM, two-tailed unpaired t-test (n = 3), *p < 0.05 and ***p < 0.001).

Immunoblotting confirmed efficient knockdown of RNF213 and we observed a strong downregulation of DDAH1 and CYR61 under these conditions (**Figure 2A** and **Supplementary Figures 3A, B**). In contrast, MVP was upregulated, especially in the presence of IFN-β, in line with our proteomics results (**Supplementary Figure 3C**). Taking into account the recently discovered interaction between RNF213 and ISG15, we also monitored the levels of ISG15. As expected, we observed that IFN-β treatment increased the levels of both free and conjugated ISG15. We next tested whether protein-level regulation of MVP, DDAH1 and CYR61 is preceded by changes at the transcriptional level and therefore measured mRNA levels by qPCR (**Figure 2B**). Knockdown of RNF213 resulted in significant upregulation of MVP in the presence of IFN and significantly lower mRNA levels for CYR61 and DDAH1 under all conditions, indicating that RNF213-mediated protein regulation results from changes at the transcriptional level.

### DDAH1 Counteracts *Listeria monocytogenes* Infection *In Vitro*

**Figure 3:**
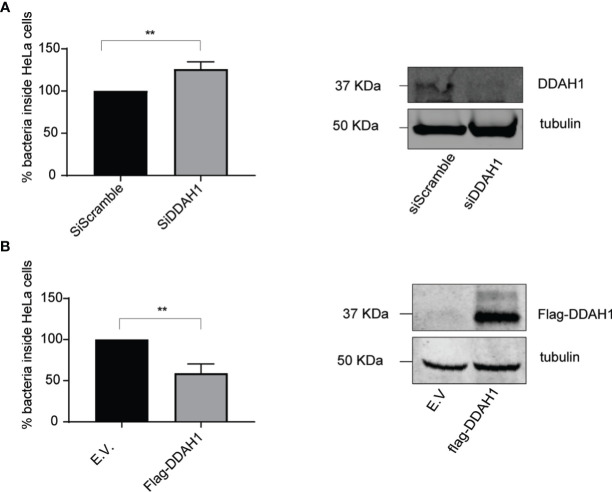
DDAH1 counteracts *Listeria* in HeLa cells. **(A)** HeLa cells were infected with *Listeria monocytogenes* EGD for 24 h at a multiplicity of infection (MOI) of 25. 24h prior to infection, cells were transfected with a pool of siRNAs targeting DDAH1 or a pool of scrambled siRNAs (siScramble) as control. Knockdown of DDAH1 led to significantly higher infection levels compared to scrambled siRNA control. Immunoblotting against DDAH1 confirmed efficient knockdown of DDAH1 with tubulin used as loading control. **(B)** HeLa cells were infected with *Listeria* EGD for 24 h at a MOI of 25. 24h prior to infection, cells were transfected with a plasmid encoding FLAG-DDAH1 or an empty vector (E.V.) as control. Overexpression of a FLAG-tagged DDAH1 significantly lowered *Listeria* infection levels. Immunoblotting against FLAG confirmed expression of FLAG-DDAH1 with tubulin used as loading control. Data represents 3 biological replicates with 3 technical replicates per condition. (AVG ± SEM, two-tailed unpaired t-test (n = 3), **p < 0.01).

Our previous discovery of RNF213 as an antimicrobial protein (Thery et al., 2021) along with the recently reported anti-bacterial activity of CYR61 (Jun and Lau, 2020) made us hypothesize that DDAH1 might also hold antimicrobial properties. To investigate this, we infected HeLa cells with reduced or enhanced expression levels of DDAH1 with *Listeria* at a multiplicity of infection (MOI) of 25, measuring the effect of CYR61 and MVP in parallel. Without any difference in bacterial entry (**Supplementary Figures 4A, B**), knockdown of DDAH1 led to significantly higher infection levels compared to the scrambled siRNA control (**Figure 3A**). Conversely, overexpression of FLAG-tagged DDAH1 significantly lowered *Listeria* infection levels (**Figure 3B**). Infecting cells at a lower MOI of 10 resulted in similar, but less pronounced significant differences (**Supplementary Figures 4C–F**). As expected, overexpression of FLAG-CYR61 also reduced infection levels, but no increase in infection was observed upon (partial) knockdown of CYR61 (**Supplementary Figures 5A, B**). Overexpression of FLAG-MVP did not affect infection levels, but knockdown resulted in a significant decrease in infection levels, in line with its documented role to protect *Listeria* from autophagy (Dortet et al., 2011) (**Supplementary Figures 5C, D**) To exclude any cell type specific effects, we repeated the experiments with DDAH1 in HCT-116 cells, an intestinal epithelial cell line relevant for *Listeria* infection (Burrack et al., 2009). While knockdown of DDAH1 in HCT-116 cells increased the bacterial load even more than in HeLa cells, both at MOI 10 and 25, overexpression did not have any effect, maybe due to saturating levels of endogenous DDAH1 in these cells (**Supplementary Figures 6A-H**). Together, these results show that DDAH1 indeed holds antimicrobial properties, counteracting replication of *Listeria* in epithelial human cells.

### RNF213 Is Required for NO Production and Control of *Listeria* in Macrophages

**Figure 4:**
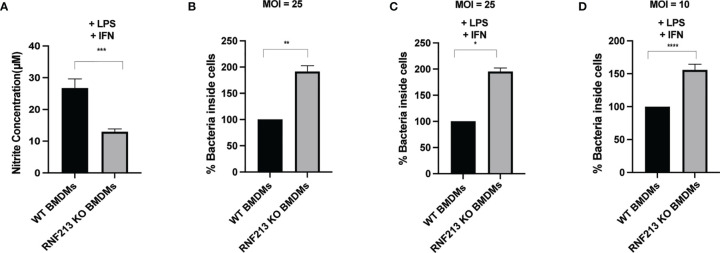
RNF213 is required for NO production and control of *Listeria* in macrophages. **(A)** BMDM cells derived from RNF213+/+ or RNF213-/- mice were treated with IFN-γ (10 ng/ml) and LPS (100 ng/ml) 24h prior to measurement of the nitrite concentration in the cell culture medium. Significantly lower nitrite levels were measured in the culture medium of RNF213-/- BMDMs compared to RNF213+/+ BMDMs. Data from a representative experiment with 8 biological replicates per condition is shown. (AVG ± SEM, two-tailed unpaired t-test (n = 8), ***p < 0.001). **(B)** BMDM cells derived from RNF213+/+ or RNF213-/- mice were infected with *Listeria* for 16h at a MOI of 25. Colony Forming Units (CFUs) were counted by serial dilution and plating showing that RNF213 -/- BMDMs are more susceptible to *Listeria* infection. Data from a representative experiment with 4 biological replicates per condition is shown. (AVG ± SEM, two-tailed unpaired t-test (n = 4), **p < 0.01). **(C, D)** BMDM cells derived from RNF213+/+ or RNF213-/- mice were treated with IFN-γ (10 ng/ml) and LPS (100 ng/ml) 24h prior to infection with *Listeria monocytogenes* EGD for 16 h at a multiplicity of infection (MOI) of 25 **(C)** or 10 **(D)**. Colony Forming Units (CFUs) were counted by serial dilution and plating showing that RNF213 -/- BMDMs are more susceptible to *Listeria* infection. Data from a representative experiment with 4 biological replicates per condition is shown. (AVG ± SEM, two-tailed unpaired t-test (n = 4), *p < 0.05, ****<0.00001).

Since knockdown of RNF213 downregulates DDAH1 (**Figure 2**) and since DDAH1 counteracts *Listeria* (**Figure 3**), we wondered whether RNF213 has any effect on cellular NO production *via* DDAH1 as a potential mechanism underlying the observed antilisterial effect. DDAH1 metabolizes methylarginines which are important endogenous inhibitors of nitric oxide synthase (NOS) (Hulin et al., 2020). Thus, reduced levels of RNF213 and DDAH1 are expected to lead to accumulation of methylarginines and lower levels of NO. To test this hypothesis, we measured NO production by bone marrow derived macrophages (BMDMs) isolated from WT or RNF213 KO mice. Upon stimulation with LPS and IFN-γ to induce NO production by inducible NOS (iNOS), we measured significantly lower nitrite levels in the culture medium of RNF213 KO BMDMs (**Figure 4A**). Interestingly, this correlated with a higher susceptibility of these cells to infection with *Listeria*, both with and without LPS and IFN-γ stimulation and at two different MOIs (**Figures 4B–D**). Moreover, these findings fit well with our previous *in vivo* data showing a dramatically increased susceptibility to *Listeria* of RNF213 deficient mice (Thery et al., 2021). Overall, our data presented here indicate a potential mechanism by which RNF213 is capable to control *Listeria* through production of NO, most likely *via* transcriptional regulation of DDAH1 expression levels.

## Discussion

Mutations in RNF213 predispose to Moyamoya disease (MMD), a rare cerebrovascular disorder. Without any link to MMD, two independent studies recently identified RNF213 as an antimicrobial protein that can bind to cytosolic *Listeria* and *Salmonella* and that is important to control infection *in vitro* and *in vivo* (Otten et al., 2021; Thery et al., 2021). While in the case of *Salmonella*, ubiquitination of LPS in the bacterial cell wall precedes clearing of the bacteria *via* xenophagy, the mechanism by which RNF213 counteracts intracellular *Listeria* is still unclear. While a similar mechanism cannot be excluded, only 7% of intracellular *Listeria* co-localizes with ubiquitin (Yoshikawa et al., 2009), whereas 40% of *Listeria* localizes with RNF213 (Thery et al., 2021), suggesting that not all RNF213 decorated *Listeria* are ubiquitinated. Therefore, the question remains whether RNF213 directly ubiquitylates components of Gram+ bacteria such as *Listeria*. Using an untargeted proteomics screen, we here report that depletion of RNF213 leads to upregulation of MVP, as well as downregulation of CYR61 and DDAH1, indicating that RNF213 also controls the expression of protective host proteins.

MVP localizes in the cytoplasm and in the perinuclear region. It is required in the regulation of several cellular processes such as immune responses (Yu et al., 2002; Yi et al., 2005; Kim et al., 2006; Steiner et al., 2006) and for normal vault structure (Scheffer et al., 1995; Suprenant, 2002; Zheng et al., 2005). Vaults are ribonucleoprotein complexes that contain multiple copies of MVP and that are likely involved in intracellular transport (Kickhoefer et al., 1999). MVP triggers production of cytokines, chemokines, and IFN‐1 during viral infection (Schoggins et al., 2011; Liu et al., 2012; Peng et al., 2016; Wang et al., 2020) and mediates resistance to *Pseudomonas aeruginosa* infection (Kowalski et al., 2007). Previously, it has been described that *Listeria* is able to decorate its surface with MVP, in order to escape autophagic recognition (Dortet et al., 2011; Dortet et al., 2012). Given that RNF213 also has the capacity to directly bind to bacteria (Otten et al., 2021; Thery et al., 2021), it is plausible that MVP and RNF213 directly compete to bind *Listeria*, with a different outcome of the fate of the bacterium. In contrast to MVP, CYR61 is a reported antibacterial protein which we found downregulated upon knockdown of RNF213. CYR61 is a constituent of the extracellular matrix, produced and secreted by several cell types. CYR61 stimulates endothelial cell growth, migration, adhesion, and survival. CYR61 is involved in angiogenesis and its action is mediated through interactions with integrins which regulate the activity and production of other angiogenic proteins (Sorokin, 2010). In previous studies, CYR61 was found to bind bacterial molecular patterns including LPS of Gram- bacteria and peptidoglycans of Gram+ bacteria, and to opsonize these bacteria for clearance (Jun and Lau, 2020). Furthermore, CYR61 can bind directly to Toll Like Receptor 2 (TLR2) and Toll Like Receptor 4 (TLR4) and activate Myeloid differentiation primary response 88 (MYD88)-dependant signalling, as well as cytokine expression and neutrophil mobilization (Jun and Lau, 2020). We found that overexpression of CYR61 slightly protected HeLa cells against *Listeria* infection. Of note, genome-wide siRNA screens in *Listeria* infected HeLa cells previously ranked CYR61 as a protective host protein, in line with our observations and intriguingly, CYR61 is ranked close to RNF213 (**Supplementary Figure 2**) (Kuhbacher et al., 2015). As with MVP, it would be interesting to evaluate potential competition, or in the case of CYR61 maybe concerted binding, with RNF213 to the surface of *Listeria*. To evaluate such spatial effects, it will however be important to take into account temporal expression of endogenous proteins, since our results indicate that MVP and CYR61 act under transcriptional control downstream of RNF213. The latter ties in well with previous studies wherein RNF213 knockdown was shown to mitigate transcription of proteins involved in the NF-κB pathway (Piccolis et al., 2019) and can regulate the transcription factor Hypoxia-inducible factor 1-alpha (HIF-1a) and α-ketoglutarate-dependent dioxygenases (α-KGDDs) (Banh et al., 2016). Overall, our results indicate that RNF213 not only post-translationally modifies (bacterial) molecules *via* its E3 module, but also exerts transcriptional control over host defence proteins. How this control is mediated and what are the transcription and other factors involved is subject for future research.

As most important finding, we identified DDAH1 as strongly downregulated protein after silencing of RNF213. DDAH1 localizes in the cytosol, but can also be secreted in the extracellular region (Leiper et al., 2007). DDAH1 hydrolyzes asymmetric dimethylarginine (ADMA) and N-Methylarginine (MMA) which act as inhibitors of nitric oxide synthase (NOS) (Zhang et al., 2011) (Leiper et al., 1999). DDAH1 has therefore an important role in the regulation of NO synthesis. NO is a transitory free radical implicated in diverse biological processes in the body, including the regulation of blood pressure, the control of platelet aggregation, and protection against vascular injury caused by tissue deposition of immune complexes. Moreover, NO is used as a broad spectrum antimicrobial agent by both the innate and cell-mediated immune systems (Nathan, 1992; Bogdan et al., 2000; Fang, 2004). We report here an antimicrobial role of DDAH1 against *Listeria* infection. Like CYR61, a genome-wide siRNA screen in *Listeria* infected HeLa cells previously ranked DDAH1 as a protective host protein (Kuhbacher et al., 2015) (**Supplementary Figure 2**). Interestingly, a previous study found that mainly inducible NOS (iNOS) is responsible for NO production during *Listeria* infection in resting BMDMs, but, somehow surprisingly, BMDMs deficient for iNOS did not show increased levels of intracellular *Listeria* (Zwaferink et al., 2008). We however found that reduced production of NO by activated BMDMs deficient for RNF213 did correlate with increased *Listeria* replication. Hence, it would be interesting to explore the link between DDAH1 and iNOS in more depth. In DDAH1 KO-/+ animals, reduced NO production was linked to endothelial dysfunction, but without evaluation of potential immune effects (Leiper et al., 2007).

Taken together, our findings that depletion of RNF213 leads to reduced levels of DDAH1 and to lower levels of NO production suggest a potential mechanism by which RNF213 controls *Listeria* infection through regulation of DDAH1 transcription. Furthermore, our study provides a springboard for further experiments on a potential role of NO in the development of MMD, especially considering that bi-allelic loss-of-function mutations in GUCY1A3, the major receptor for NO, cause an autosomal recessive form of MMD (MIM: 615750) (Bryan et al., 2009). Notably, in a small cohort study it was shown that NO metabolites were elevated in the cerebral spinal fluid (CSF) of MMD patients, possibly reflecting development of impaired circulation (Noda et al., 2000). Given the important role of NO in both vascular and immune function, it would be most interesting to further investigate a potential role of NO in MMD pathogenesis.

## Data Availability Statement

The proteomics data have been deposited in the PRIDE database under accession code PXD027130.

## Ethics Statement

Bone marrow-derived macrophages were isolated from animals housed in an animal facility that operates under the Flemish Government License Number LA1400536. All experiments were done under conditions specified by law and authorized by the UGent Institutional Ethical Committee on Experimental Animals.

## Author Contributions

LM, CA, and FI conceived and planned the experiments. LM and CA carried out the experiments. LD and SE provided support on the qPCR analysis. FT and TM provided support on proteomics data analysis. LM and CA took the lead in writing the manuscript. All authors provided critical feedback in addition to help shape the research, data-analysis and manuscript. All authors contributed to the article and approved the submitted version.

## Funding

FI acknowledges funding from Odysseus grant G0F8616N from the Research Foundation Flanders (FWO) and ERANET Infect-ERA BacVIRISG15. LD was supported by a FWO-SB fellowship and by a BOF-GOA grant (2016000602 to SE). BD is supported by an Odysseus type 1 Grant of the Research Foundation Flanders (3G0H8318) and a starting grant from Ghent University Special Research Fund (01N10319).

## Conflict of Interest

The authors declare that the research was conducted in the absence of any commercial or financial relationships that could be construed as a potential conflict of interest.

## Publisher’s Note

All claims expressed in this article are solely those of the authors and do not necessarily represent those of their affiliated organizations, or those of the publisher, the editors and the reviewers. Any product that may be evaluated in this article, or claim that may be made by its manufacturer, is not guaranteed or endorsed by the publisher.
