# Supplementary material for: Proteome Profiling of RNF213 Depleted Cells Reveals Nitric Oxide Regulator DDAH1 Antilisterial Activity
Source: Front Cell Infect Microbiol. 2021 Nov 3;11:735416. doi: 10.3389/fcimb.2021.735416 (PMC8595287; doi:10.3389/fcimb.2021.735416)
Supplement: Supplementary file 1 [file DataSheet_1.docx]

Supplementary Figures

**Supplementary Figure 1**

**Supplementary Figure 1:** **RNF213 depleted cells correctly express ISGs.** **(A-B)** Volcano plots showing the result of Perseus t-tests (FDR=0.05; S0=1) comparing IFNβ treated versus untreated cells, either in the presence or absence of RNF213 knockdown. The fold change (in log_2_) of each protein is shown on the X-axis, while the statistical significance (−log P-value) is shown on the Y-axis. Proteins outside the curved lines represent differentially regulated proteins upon RNF213 depletion (Supplementary Table S3 and S4). **(C)** Proteins differentially regulated in the above volcano plots were displayed on a heat map after non-supervised hierarchical clustering. On the right side, the same heat map is shown with originally missing values colored in gray. **(D)** Profile blots showing protein intensities (log_2_ LFQ values) in the different samples of a selected number of ISGs: ISG15, STAT1, MXA and IFIT1.

**Supplementary Figure 2**

**Supplementary Figure 2:** **DDAH1 and CYR61 ranked as protective host factors in a previous genome-wide siRNA screen** (Kuhbacher, 2015). Distribution of Z scores of infection determined by knockdown of roughly 16,000 host genes in combination with *Listeria* infection in HeLa cells, taken from (Kühbacher et al., 2014). Positive values are associated with infection-increasing knockdown, while negative values are associated with infection-reducing knockdown. In this screen, CYR61 and DDAH1 ranked as protective host factors, close to RNF213. Remarkably, also MVP classified as protective host factor in this screen, in contrast to the findings of Dortet et al. (Dortet et al., 2012) and the observations in the present study (Supplementary Figure 5).

 **Supplementary Figure 3**

**Supplementary Figure 3: Densitometric western blot analysis of DDAH1, CYR61 and MVP.** **(A-C).**  The experiment shown in Figure 2A was repeated two more times to allow densitometry and statistical comparison using the data from all three biological repeats. Briefly, HeLa cells were transfected with a pool of scrambled siRNAs (siScramble) or siRNA targeting RNF213 (siRNF213), and treated with IFN- ß for 24h or left untreated. Immunoblotting against DDAH1 confirmed significant downregulation of DDAH1 (A) and CYR61 (B) and upregulation of MVP (C) upon knockdown of RNF213. Data represents 3 biological replicates, AVG± SD, two- tailed Student t-test (n=3). A representative western blot is shown in Figure 2A. Asterisks indicate p values with *p < 0.05, **p < 0.01, ***< 0.001.

**Supplementary Figure 4**

**Supplementary Figure 4: Antilisterial effect of DDAH1.** **(A-F)** HeLa cells were infected with *Listeria monocytogenes* EGD for 1h at a multiplicity of infection (MOI) of 25 (A, B) or for 24h at a MOI of 10 (C, D). 24 h prior to infection, cells were transfected with a pool of siRNAs targeting DDAH1, a pool of scrambled siRNAs (siScramble) as control (A, C, E), a plasmid encoding FLAG-DDAH1 or an empty vector (E.V.) as control (B, D, F). After 1h of infection at a MOI of 25, no difference in CFUs was detected in cells with silencing or overexpression of DDAH1 compared to their respective controls (A, B). After 24h of infection at an MOI of 10, silencing of DDAH1 led to significantly more bacteria in the cells (C), whereas overexpression of DDAH1 led to a reduction of intracellular bacteria (D). Immunoblots against DDAH1 (E) and FLAG (F) confirmed efficient knockdown of DDAH1 and expression of FLAG-DDAH1, respectively, using tubulin as loading control. Data represents 3 biological replicates, AVG± SEM, two- tailed Student t-test (n=3). Asterisks indicate p values with *p < 0.05, **p < 0.01).

**Supplementary Figure 5**

**Supplementary Figure 5**: **Pro- and antilisterial effects of MVP and CYR61.** **(A, B)** HeLa cells were infected with *Listeria monocytogenes* EGD for 24 h at a multiplicity of infection (MOI) of 25. 24 h prior to infection, cells were transfected with a pool of siRNAs targeting CYR61, a pool of scrambled siRNAs (siScramble) as control (A), FLAG-CYR61 or empty vector (E.V.) as control (B). While knockdown of CYR61 did not lead to a significant difference compared to the scrambled siRNA control, overexpression of FLAG-CYR61 did lead to a significant reduction in infection. (C, D) HeLa cells were infected with *Listeria monocytogenes* EGD for 24 h at a multiplicity of infection (MOI) of 25. 24 h prior to infection, cells were transfected with a pool of siRNAs targeting MVP, a pool of scrambled siRNAs (siScramble) as control (C), FLAG-MVP or empty vector (E.V.) as control (D). Knockdown of MVP led to significantly reduced infection levels, while overexpression of FLAG-MVP did not lead to a significant difference in infection level. (A-D) Immunoblots against MVP and CYR61 confirmed efficient knockdown of MVP and partial knockdown of CYR61, using tubulin as loading control. Immunoblots against FLAG confirmed efficient expression of FLAG-MVP and FLAG-CYR61, using tubulin as loading control. Data represents 3 biological replicates, AVG± SEM, two- tailed Student t-test (n=3). The results a representative experiment is shown in Figure 2. Asterisks indicate p values with *p < 0.05, **p < 0.01).

**Supplementary Figure 6**

**Supplementary Figure 6: Antilisterial effect of DDAH1.** **(A-F)** HCT116 cells were infected with *Listeria monocytogenes* EGD for 1h at a multiplicity of infection (MOI) of 25 (A, B), for 24h at a MOI of 25 (C, D) or for 24h at a MOI of 10 (E, F). 24 h prior to infection, cells were transfected with a pool of siRNAs targeting DDAH1, a pool of scrambled siRNAs (siScramble) as control (A, C, E, G), a plasmid encoding FLAG-DDAH1 or an empty vector (E.V.) as control (B, D, F, H). After 1h of infection at a MOI of 25, no difference in CFUs was detected in cells with silencing or overexpression of DDAH1 compared to their respective controls (A, B). After 24h of infection, silencing of DDAH1 led to significantly more bacteria in cells infected with a MOI of 25 (C) as well as a MOI of 10 (E). In contrast, overexpression of DDAH1 did not lead to a significant reduction of bacteria in the HCT-116 cells (D, F). Immunoblots against DDAH1 (G) and FLAG (H) confirmed efficient knockdown of DDAH1 and expression of FLAG-DDAH1, respectively, using tubulin as loading control. Data represents 2 biological replicates, AVG± SEM, two- tailed Student t-test (n=2). Asterisks indicate p values with ***p < 0.001).
